# Influence of Age and Individual Differences on Mouthfeel Perception of Whey Protein-Fortified Products: A Review

**DOI:** 10.3390/foods10020433

**Published:** 2021-02-16

**Authors:** Victoria Norton, Stella Lignou, Lisa Methven

**Affiliations:** Department of Food and Nutritional Sciences, Harry Nursten Building, University of Reading, Whiteknights, Reading RG6 6DZ, UK; v.l.norton@pgr.reading.ac.uk (V.N.); s.lignou@reading.ac.uk (S.L.)

**Keywords:** older adults, individual differences, whey protein, mouthdrying, protein-fortified products

## Abstract

Protein needs are considered to increase with age, with protein consumption being associated with many positive outcomes. Protein-fortified products are often used to improve nutritional status and prevent age-related muscle mass loss in older adults. Accordingly, older adults are commonly provided with products fortified with whey protein; however, such products can cause mouthdrying, limiting consumption and product enjoyment. Currently, the extent to which age and individual differences (e.g., saliva, oral health, food oral processing) influence the perception of whey protein-derived mouthdrying is relatively unclear. Previous research in this area has mainly focused on investigating mouthdrying, without taking into account individual differences that could influence this perception within the target population. Therefore, the main focus of this review is to provide an overview of the relevant individual differences likely to influence mouthfeel perception (specifically mouthdrying) from whey protein-fortified products, thereby enabling the future design of such products to incorporate better the needs of older adults and improve their nutritional status. This review concludes that age and individual differences are likely to influence mouthdrying sensations from whey protein-fortified products. Future research should focus more on the target population and individual differences to maximise the benefits from whey protein fortification.

## 1. Introduction to Malnutrition in Older Adults

In recent decades, there has been a worldwide increase in ageing populations and in 2019, globally, there were 703 million individuals aged 65 years or over; this is predicted to increase to 1.5 billion by 2050 [1]. Older adults are typically described as people aged 65 years or over [1,2], and within this review, older adults will be referred to as individuals aged 65 years or over. However, this description reflects a broad range of individuals with differing needs and abilities. Ageing can be described simply as getting older and more specifically, from a biological viewpoint, as the accumulation of molecular and cellular damage over a lifespan contributing to a decline in function and increased disease risk [3]. The health needs of an ageing population can, however, be described as complex and associated with physiological changes, disease and multimorbidity [3]. The World Health Organisation (WHO) have used the term ‘healthy ageing’ to promote functional ability within an ageing population and more recently introduced 2020–2030 as the ‘Decade of Healthy Ageing’ to provide a focus on improving the lives of older adults [4]. In addition, simple health behaviours, such as good nutrition and physical activity, can provide health and well-being benefits, as well as promoting longevity [3].

Good nutrition is associated with numerous positive benefits, such as improved health and well-being. Energy, protein, vitamin C, vitamin D, folate, iron, zinc and fibre are considered important nutrients for older adults [5]. Energy requirements are considered to decline with age due to body composition changes and reduced physical activity [6]. However, protein needs are considered to increase with age (as outlined in Section 2), but, as with most other nutrients, recommendations typically remain the same as those suggested for adults generally [7,8,9] (for a recent review on nutritional recommendations in older adults see Dorrington et al. [10]). Additionally, there are adverse effects associated with the ageing process, which is considered to be a multidimensional process, including physical, psychological and social changes, all of which are potential risk factors for malnutrition (Table 1) [11].

Varying definitions of malnutrition are reported within the literature [15]. One of the most commonly used describes malnutrition as a “deficiency or excess (or imbalance) of energy, protein and other nutrients” resulting in negative consequences “on tissue/body form (body shape, size and composition) and function and clinical outcome” [13]. Typically, the focus within older adults is largely on undernutrition (a deficiency in both macronutrients and micronutrients) [13,16]. Malnutrition is prevalent amongst older adults with increased risks associated with age, gender (female), disease status and clinical settings (hospitals, care homes and mental health units) [13,17]. Twenty-three percent of European older adults are considered at risk of malnutrition and over one million older adults are affected in the UK [13,17]. Malnutrition is commonly linked with reduced functional status, muscle function, bone mass and cognitive function, poor wound healing, delayed recovery from surgery, mortality and higher hospital readmission rates [11]. A five-step screening process ‘Malnutrition Universal Screening Tool’ (MUST) is regularly used in the UK to categorise patients for risk of malnutrition in a range of clinical settings [18]. Typically, an individual can be described as ‘malnourished’ if they have a body mass index (BMI) lower than 18.5 kg/m^2^ or an unplanned weight loss (>10% within the last 3–6 months) or a combination of a BMI lower than 20 kg/m^2^ and an unplanned weight loss (>5% within the last 3–6 months) [19].

Malnutrition can contribute to sarcopenia, which is an age-related loss of skeletal muscle mass and function that is exacerbated by low protein intake alongside poor conversion of protein to muscle mass [20]. Sarcopenia has been reported to affect 5–13% of adults in their sixties and 11–50% of those in their eighties [21]. Despite it being considered a preventable and treatable condition, it is a contributor to increasing health costs; muscle weakness conditions in the UK cost £2.5 billion per annum [20,22]. Sarcopenia has been identified as a potential precursor to frailty, both conditions being multi-dimensional, reversible and with inflammatory links [23]. Frailty can lead to reduced strength, endurance and physiological function, thereby increasing vulnerability to external stressors [24].

Reduced appetite can lead to increased risk of malnutrition in older adults. Factors such as loss of smell, oral and taste impairments, medication, anorexia of ageing, physiological, psychological and social factors can all contribute to a decline in appetite [25,26,27,28,29]. Additionally, this decline is considered to result partially from delayed gastric emptying (increased time food spends in the stomach), thereby increasing satiation and reducing appetite [30]. The type of foods (liquid or solid) consumed can influence food intake. For example, in a study involving healthy older adults consuming a liquid beverage resulted in increased subsequent intake (13.4% increase in oatmeal) compared with a solid energy bar [31]. Furthermore, the texture of foods (such as chewy, hard and viscous) influence appetite regulation, where increased processing within the mouth has been shown to lead to increased feelings of satiety [32]. A meta-analysis by Giezenaar et al. highlighted reduction in energy intake, hunger and increased fullness were all associated with age [33], therefore, promoting foods that encourage food intake is key to counterbalancing this.

Poor oral health can have a detrimental impact on an individual’s nutritional status, health and well-being [34]. Oral impairments can impact biting, chewing and swallowing of foods [35]. Older adults typically suffer from teeth loss, dental caries, reduced saliva flow, changes in oral mucous membrane and chewing efficiency, mouth dryness and increased periodontal diseases and use of dentures, all likely to influence food habits and intake [34]. For example, data from the UK National Diet and Nutrition Survey 2008–2014 identified that dental status impacted food selection, and nutrient intake in older adults with compromised dental status (such as edentate and/or dentate with denture wearers) had a negative effect on intake [36]. Kremer et al. demonstrated that older adults, who were denture wearers, perceived custards to be less creamy and less easy to swallow compared with those with natural teeth [37]. Dentures can result in changes in mouth movements, chewing efficiency and sensory thresholds [35,37,38]. Saliva lubrication can influence comfort of wearing dentures [35] and decline in oral health can also contribute to taste disorders within older adults [39]. For example, poor oral hygiene, dry mouth, caries and high growth of oral bacteria have been shown to decrease taste ability in acutely hospitalised older adults (70–103 years) [40]. Therefore, maintaining good oral health can increase appetite, food intake and improve taste perception [40]. The impact of medication must also be considered when investigating nutritional status and age. A recent Health Survey for England (2016) identified that whereas only 19% of young adults (16–24 years) used at least one prescription medication per week, this increased to 80% of older adults (65–74 years) and was 96% for those over 85 years [41]. Medication commonly has side effects such as affecting oral health, appetite and taste [29,42], thereby contributing to an increased risk of poor nutritional status.

**Figure 1:**
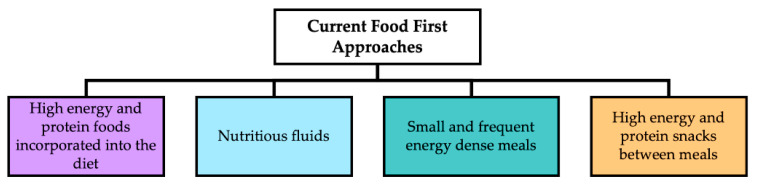
Commonly used strategies to improve nutritional intake in older adults at risk of malnutrition (adapted from [12]).

Nutritional support can provide a cost-effective treatment to improve functional and clinical outcomes for individuals at risk of malnutrition [43]. Accordingly, the British Dietetic Association (BDA) promotes a food first approach to enhance nutritional intake; recommended strategies are outlined in Figure 1 [12].

In summary, the health and nutritional needs of an ageing population are considered complex and involve numerous age-related changes, which subsequently influence food intake and quality of life, all contributory factors to poor nutritional status amongst older adults. Protein-fortified products provide a key role in promoting a food-first approach to enhance nutritional intake and support the recognised increased protein needs with age. Such products often contain animal-derived proteins, for example whey proteins, which are considered complete sources of protein (including all essential amino acids), whereas plant-derived proteins are typically incomplete sources of protein (lacking one or two essential amino acids) [44]. Furthermore, animal-derived proteins are more readily digestible and effective in muscle protein synthesis than plant-derived proteins [44]. It is, therefore, important that whey protein-fortified products have consumer acceptance, which relies on a good sensory profile. However, recent reviews [45,46] have shown such products are associated with astringency or mouthdrying attributes. To date, such reviews have not considered the additional dimension of individual differences within the target population. Establishing the impact that age and individual differences may have on whey protein perception would have particular relevance for older adults so as to mitigate characteristics linked to poor consumer acceptance. Accordingly, the main aims of this review are to summarise the latest research relating to (a) protein-fortified products for older adults; (b) exploration of the mechanisms underpinning whey protein-derived mouthdrying; (c) the influence that age and individual differences could have on the perception of whey protein-derived mouthdrying, as well as providing suggestions for future research.

## 2. Protein Requirements and the Importance of Protein-Fortified Products in the Diet of Older Adults

Proteins are polymers of amino acids and provide key roles in tissue growth and repair [47]. The ‘Protein for Life’ research team has recently identified that many individuals within the UK have inadequate protein intake to maintain muscle strength and function in older age [22]. In addition, an improvement in protein intake during the life course could potentially reduce the onset of certain health conditions and also slow the rate of muscle decline [48]. The ‘Protein for Life’ focus groups, which involved healthy adults, also identified a lack of certainty over optimal protein intakes during the life course [22]. The UK current reference nutrient intake (RNI) for adults is 0.75 g/kg/d [47], yet protein needs are considered to increase with age. For example, recently, the PROT-AGE study group and the European Society for Clinical Nutrition and Metabolism (ESPEN) expert group have both reviewed protein intake in the older population [7,8]. Both studies have recommended a protein intake of 1.0–1.2 g/kg/d for older adults and higher protein intakes (1.2–1.5 g/kg/d) for older adults suffering from acute and chronic disease [7,8]. These findings are also supported by recent BDA and Parenteral and Enteral Nutritional Group (PENG) guidelines for nutritionally vulnerable adults in clinical settings, which recommended protein intake of 1.1 g/kg/d [12,49]. These increased protein intakes are considered necessary to maintain good health, encourage recovery from illness and preserve functionality as a result of age-related changes in protein metabolism [7]. In addition, factors such as sarcopenia, anabolic resistance, disease related protein catabolism, low postprandial amino acid availability and decreased muscle perfusion can also result in increased protein needs for an older adult [8]. The ESPEN Expert Group identified various possible causes for reduced protein intake in older adults, including socioeconomic status, medical conditions, physiological changes, genetic predisposition and physical disability [8].

Protein is considered a satiating macronutrient which can lead to reduced intake at subsequent meals compared with fat and carbohydrates [50]. The proposed mechanisms include increased diet induced energy expenditure, satiety hormones and amino acids, as well as modulation of gluconeogenesis [50]. However, such studies have generally been in younger adults and the response may be modulated by age [51,52]. For example, in two studies by Giezenaar et al. whey protein drinks were not found to be satiating in older adults compared with younger adults [51,52]. They identified older male volunteers showing an increase in appetite, slower gastric emptying and increased overall energy intake [51]. However, appetite decreased following consumption of whey protein drinks in younger male volunteers [51]. Additionally, with older male and female volunteers, *ad libitum* energy intake was not affected 3 h post whey protein drink consumption [52]. Appetite is considered to decrease with age per se, and these findings provide support for protein supplementation as an effective nutritional intervention to increase protein intake.

In order to enhance nutritional intake in older adults, a product needs to be palatable, appetising, of suitable portion size and energy dense [30]. Typically, oral nutritional supplements (ONS) and protein-fortified products are used to improve protein intake in older adults. ONS are commonly consumed by older adults and those at risk of malnutrition, where they are unable to meet nutritional needs from their diet [53]. They consist of products which provide macro and micronutrients in semi-solid, powder or liquid form [53]. Protein powders have varied applications and uses within food processing [54] and many high energy drinks contain whey protein (further outlined in Section 3) due to its high nutritional and functional values [51,55] often as whey protein isolate (WPI) or whey protein concentrate (WPC) [56]. Protein-fortified meals and snacks can provide a simple alternative to ONS and provide familiar foods to older adults which can encourage consumption by increasing energy and protein intake [57,58]. Variety is required to avoid taste fatigue and improve compliance and intake amongst older adults, including different flavours, textures and appearance [30].

Multiple studies have demonstrated benefits from protein supplementation and/or protein fortification. For example, Cawood et al. carried out a systematic review of 36 studies highlighting benefits of high protein ONS (20–54% energy from protein) and concluded there was a 19% reduction in complications (healing of surgical wounds, pressure ulcers and infections rates) following consumption across various settings (hospital and community settings) [59]. A recent randomised control trial was carried out with 104 malnourished care-home residents comparing ONS outcomes (*n* = 53) with dietary advice (*n* = 51) for 12 weeks [60]. The ONS had energy density between 1.3–4.5 kcal/mL with voluntary intake measured against a target of 600 kcal and 16 g protein per day [60]. This study supported that nutritional intake and quality of life were significantly improved in the ONS group compared with the conventional dietary advice group [60]. Bauer et al. demonstrated a significant improvement in muscle mass following a three-month period of ONS consumption, containing vitamin D and leucine-enriched whey protein, compared with the control group, amongst older adults with sarcopenia [61].

Food fortification using familiar foods could also be considered as a viable route to increase protein intake within an ageing population. A study involving a hospital setting compared the provision of protein-fortified meals (23 dishes fortified with milk protein 6.1 to 11.5 g of protein per dish; breakfast, soups, fish, meat, side dishes and desserts) with the standard hospital menu (3 main meals with 2–3 in-between meals) [62]. The fortified food service resulted in significant improvement in protein intake amongst patients at nutritional risk [62]. Appleton and Smith noted that using improved visual cues, recognisable foods and/or identification labels can enhance liking for flavours of drinks in older adults [63]. Beelen et al. carried out a pilot study using familiar products (bread, soups, fruit juices and instant mashed potato) enriched with 5.6 to 10 g protein (dairy and soy) per portion [64]. This pilot study highlighted the benefits of such products in increasing protein intake within an older population (*n* = 22) in a clinical setting [64]. The same research group carried out a subsequent randomised controlled trial using similar protein enriched familiar products (bread, cakes, soups, porridge, meat, mashed potatoes, ice cream, fruit juice and dairy products; protein content per portion varying from 5.8 to 21.6 g) [65]. They demonstrated an increase in protein intake over a 12-week period in older adults (*n* = 75), resulting in 72% of the individuals meeting the recommended intake of 1.2 g/kg/d, whereas only 31% of those in the control group met those recommendations [65].

Negative outcomes have been associated with a high protein intake. For example, its effects on kidney function, though a recent meta-analysis demonstrated that in healthy adults this was not the case [66]. Similarly, it has also been suggested it could have a negative outcome on the gut microbiota. However, a recent randomised control trial in older men demonstrated that despite consuming 1.6 g/kg/d for 10 weeks, the gut microbiota composition and microbiota derived volatile organic compounds production remained unaltered [67]. In addition, Stratton and Elia [68] noted that minimal gastrointestinal symptoms (such as nausea, bloating and diarrhoea) could arise from ONS consumption; however, they also highlighted that there are limited studies which evaluate fully gastrointestinal tolerance. Moreover, such side effects from protein supplementation typically result from the non-protein components (e.g., lactose intolerance) [69].

In summary, protein needs are considered to increase with age, with increased protein intake associated with many positive functional outcomes. Hence, ONS and protein-fortified products prove beneficial to nutritional status.

## 3. The Use of Whey Protein to Fortify Foods for Older Adults

Bovine milk is commonly incorporated into human diets with its associated nutritional and functional benefits [70]. Milk typically comprises water, lipids, lactose (sugar) and protein, as well as minor components (such as minerals (notably calcium), vitamins (both water- and fat-soluble vitamins), hormones, enzymes and miscellaneous compounds) [71]. Milk protein mainly derives from casein (phosphoproteins; 80% of milk proteins and insoluble at pH 4.6) and whey (globular proteins; 20% of milk proteins and soluble at pH 4.6), as well as proteinaceous materials (proteose peptone (PPs) and non-protein nitrogen (NPN)) [71,72]. Whey is a by-product of cheese making; it is the liquid remaining once the milk has been coagulated (curdled) [73]. Liquid whey can be dried to produce different whey powders (see Bansal and Bhandari [73] for an extensive overview). In summary, WPI (>90% protein) is typically subjected to further processing compared with WPC (34–89% protein), resulting in its higher protein concentration and lower fat, ash (mineral) and lactose content [44,55]. Demineralised whey powder is a reduced minerals whey powder, associated with reduced corresponding tastes such as salty and bitter [73]. Whey permeate is a by-product of whey production and is a deproteinised whey powder comprising predominantly of lactose and minerals [74].

**Figure 2:**
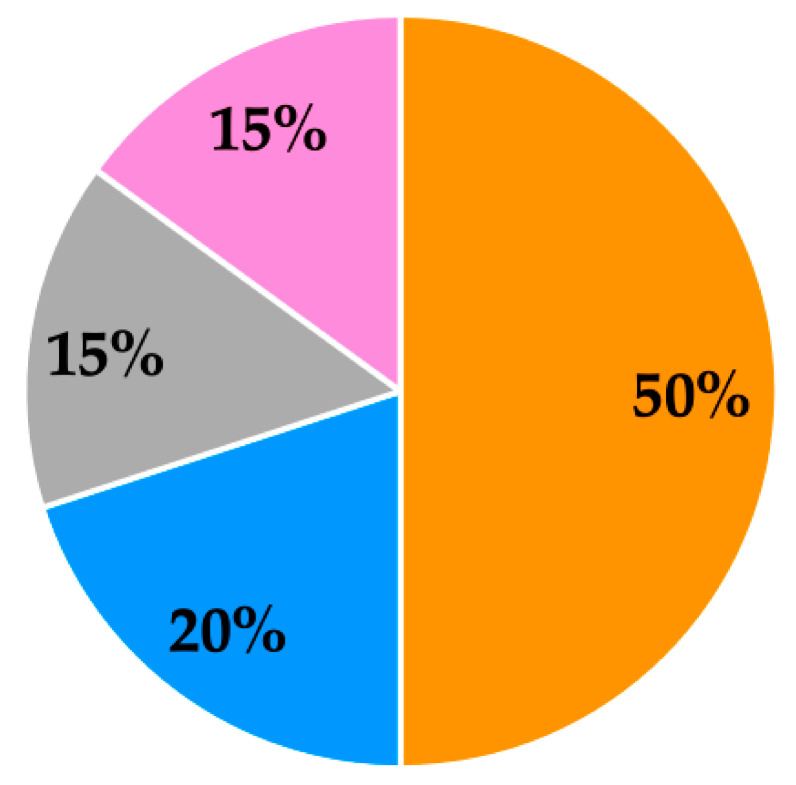
Overview of whey protein typical percentage composition [76] (minor components of whey protein are bovine serum albumin (BSA), immunoglobulins, lactoferrin and lactoperoxidase).

Whey proteins consist of β-lactoglobulin, α-lactalbumin, glycomacropeptide, bovine serum albumin (BSA), immunoglobulins, lactoferrin and lactoperoxidase in varying amounts, as summarised in Figure 2 [75,76]. Whey proteins provide a source of essential amino acids (EAAs) and branched chain amino acids (BCAAs; leucine, isoleucine and valine) [44]. In addition, whey protein is a rapidly digestible protein that is considered to provide greater nutritional benefits to older adults compared with other protein sources (such as casein), which leads to its frequent use in clinical nutritional products [77,78]. For example, the benefits of whey protein have been identified in an acute study with an older male population where postprandial muscle protein accretion was found to be more effectively stimulated by whey protein, compared with casein and casein hydrolysate [79]. Whey protein ingestion can result in an improved muscle protein synthetic response, which is considered to be due to its higher leucine content and quicker digestion and absorption kinetics compared with other protein sources [79]. Review papers have identified a number of additional potential health benefits associated with whey protein consumption, such as its antimicrobial, antiviral and anticarcinogenic effects, as well as improved immune, bone and cardiovascular health [72,80].

In order for ONS and protein-fortified products to lead to beneficial nutritional and health outcomes, enough product should be consumed to meet an individual’s daily nutritional requirements. However, compliance is reported to be variable, reducing the nutritional impact, in addition to cost and waste implications [81]. For example, a systematic review from 46 studies identified ONS compliance levels varying between 37% and 100% (with average compliance at 78%) within different settings (hospital setting: 67% and community setting: 81%) [82].

Whey protein-fortified products typically have poor consumer acceptance and this has been linked to both undesirable taste and aroma attributes, as well as negative mouthfeel attributes, such as a build-up of mouthdrying, mouthcoating, chalky, metallic and filming, associated with repeated consumption [81,83,84,85,86,87,88]. Mouthdrying has been perceived by consumers in two different whey protein-fortified food matrices [89,90]. For example, within a liquid model, beverages fortified with whey protein were associated with mouthdrying, low liking scores and presence of off flavours [83,89,91,92]. Within a solid model, snacks (such as cakes, muffins, biscuits and rye bread) fortified with whey protein were perceived as mouthdrying and/or had a dry texture and reduced liking [90,93,94]. These studies demonstrate consumers can perceive negative sensory attributes associated with whey protein-fortified products and mitigating such attributes may be the key to promoting compliance and suitability for older adults.

The sensory profile (measured using trained sensory panels) of whey proteins typically includes attributes such as aroma intensity, sweet aromatic, musty, cooked/milky, doughy/fatty (described as “aroma associated with canned biscuit dough”), metallic, cucumber, cabbage, brothy, cardboard/wet paper, animal/wet dog, pasta water, soapy, faecal, catty, grainy, opacity, bitter, astringent, chalky, thick, mouthdrying, mouthcoating, furring and body [86,95,96,97,98]. WPI and WPC are considered to have relatively similar sensory profiles (despite processing differences), with the following key differences: WPI has been shown to elicit attributes such as soapy, animal/wet dog, cucumber and bitter, which are typically not present in WPC, whereas WPC has attributes such as sweet aromatic and cooked/milky, which are not present in WPI [95].

Whey proteins are commonly fortified into a range of food matrices, with differing effects on the sensory profile. For example, trained sensory panels identified mouthfeel attributes such as chalky, drying, mouthcoating, astringency, furring and body following whey protein beverage (WPB) consumption and heat treatment of WPB is considered to intensify further these sensory properties [86]. The addition of WPI to sauces has been found to contribute additional flavour attributes (fishy, vegetable soup, chemical, savoury, bitter) as well as mouthfeel (grainy) [99]. Similarly, fortification of biscuits with WPI has been shown to alter appearance (roughness, density), flavour (bitter, savoury, burnt sugar, off flavours) and mouthfeel (teeth packing and slower melt rate) [90,100]. Cakes have also been fortified with WPI and WPC, which led to an increase in negative attributes such as mouthdrying, chewy, increased crumb size and firmness of bite [90].

In summary, whey protein fortification is a commonly used to help prevent age-related muscle mass losses. However, negative sensory attributes leading to poor consumer acceptance and compliance are commonly associated with ONS and protein-fortified products. Whey proteins are frequently cited as being a source of mouthdrying in a range of different whey protein-fortified food matrices. We consider that this needs further investigation, given that older adults are noted to suffer commonly from dry mouth or reduced saliva flow [101,102].

## 4. Mouthfeel and Mouthdrying Perception of Whey Protein-Fortified Products

Texture is considered a dynamic process as foods are continuously being manipulated within the mouth [103] and is more specifically defined as “the sensory and functional manifestation of the structural, mechanical and surface properties of foods detected through the sense of vision, hearing, touch and kinesthetics” [104]. Szczesniak and Kahn proposed consumers’ awareness of texture is increased if expectations are not met, therefore suggesting texture provides a key role in food preference [105]. Szczesniak described texture as a ‘sensory property’ and is considered best described and perceived by humans mainly via touch and pressure senses within the mouth during food evaluation [104]. Mouthfeel can be described as “the tactile (feel) properties perceived from the time at which solid, semi-solid or liquid foods or beverages are placed in the mouth until they are swallowed” [103].

Astringency, oral drying and mouthdrying are commonly used terms (and are often used interchangeably) to describe this considered ‘textural defect’ associated with dairy products [106]. The term astringency has been defined as “the complex of sensations due to shrinking, drawing or puckering of the epithelium as a result of exposure to substances such as alums or tannins” [107]. Such a perceived texture change within the oral cavity usually results from the consumption of plant-derived products rich in polyphenols, such as tea, wine, nuts and fruit [108,109]. As highlighted in recent reviews, astringency is considered a ‘complex sensation’ and potentially derived from multiple mechanisms and often builds and persists post consumption [45,110,111]. Plant-derived protein beverages (fortified with pea and soy protein) have been shown to impart astringency sensations; however, this is proposed to result from their polyphenol content rather than as a direct result of the protein composition [98,112,113]. Polyphenols are considered to interact with salivary proteins causing aggregation and precipitation, thereby reducing lubrication of saliva, increasing friction and potentially exposing mechanoreceptors, resulting in an astringent sensation [110,114,115,116]. However, as polyphenolic compounds are not present in whey protein sources, the term mouthdrying (a drying sensation in the mouth during or after consumption of a product) is considered more suitable in the context of dairy products. Accordingly, this review uses the term mouthdrying to describe whey protein-derived mouthdrying. However, astringency related oral drying from food models has been researched widely (see reviews [45,110,111]) compared with whey protein-derived mouthdrying and could, therefore, provide suggestions in terms of mechanisms, mitigating strategies and testing methods.

A recent review by Pires et al. highlighted factors such as pH, temperature, saliva, viscosity and polysaccharides as being likely to influence astringency perception [45]. Furthermore, the detection thresholds for individuals vary for astringent stimuli, which are perhaps influenced by differences in the number of receptors [111,117,118]. This has been related to indirect markers (such as 6-n-propylthiouracil (PROP status) and fungiform papillae density), as well as to direct measures of variation in oral tactile sensitivity and saliva flow [111,117,118]. A link has also been proposed between individual salivary protein content (pre- and post-stimulation) and astringency ratings in liquid food models (juices with added tannic acid and aqueous solutions with tannic acid and alum). Individuals grouped as ‘high responders’ (showing reduced replenishment of salivary proteins) perceived astringency as more intense [119,120]. However, in a solid chocolate model, differences in salivary protein were not related to perception of astringency [120]. The high fat level in the chocolate may have increased lubricity, which perhaps negated the effect of differences in salivary protein content on the perception of astringency [120].

Understanding oral movements, and where in the oral cavity volunteers perceive drying sensations, could also provide useful insights. Breslin et al. proposed astringency sensations could occur from altered and increased mechanoreceptor activity and demonstrated astringency perception was more apparent with oral movements [109]. A lack of tongue movements minimised perceived astringency in one study, suggesting astringency perception requires at least some oral movement [121]. Astringency can also be perceived on the upper lip and gum [109], suggesting a whole mouth approach is best to understand perceived astringency sensations. A key limitation within this area is the inability to measure astringency effectively. Currently, no method has been developed to achieve this, though typically, a combined approach of direct and indirect methods is used, as highlighted in a recent review [45].

### 4.1. Whey Protein-Derived Mouthdrying

Consuming dairy products can also result in a perceived texture change within the oral cavity similar to that with plant-derived products [108]. More specially, whey proteins have been shown to be a source of mouthdrying in fortified products and ONS; hence, addressing the potential causes of whey protein-derived mouthdrying is a key priority [122]. Proposed causes are summarised in Table 2. Initial research suggested that the low pH associated with some WPBs can cause mouthdrying due to protein precipitation in the mouth and subsequent saliva protein interactions [123,124,125,126,127]. The resulting mouthdrying could be related to increased particle size and turbidity [124,126]. Particle size also increases with heating time [86] and elicits a mouthdrying response at a neutral pH WPB [86,122]. Mouthdrying could also be influenced by disruption of the salivary structure causing reduced lubrication from saliva and resulting in increased friction and perceived mouthdrying [128]. There is evidence that whey proteins, a natural polymer, demonstrate tissue adhesion [129] and mucoadhesion properties [130]. For example, a previous in vitro study has shown that, despite being washed with artificial saliva, proteins remained on the buccal mucosa or tongue apex (with proteins bound to the oral mucosa) [131]. Indeed, more recently, our research group confirmed in a human oral retention study that protein does adhere to the oral cavity post WPB consumption to a greater extent [89]. Although whey proteins have a high nutritional value, they become unstable when heated, resulting in protein denaturation and aggregation, which influences the structure and stability of the protein [132]. Heat treatment of whey proteins can result in increased mouthdrying [86,133] and Bull et al. demonstrated increased oral retention of whey protein following a heated WPB compared with an unheated WPB, therefore suggesting oral retention could have a role in mouthdrying [134]. The increased mucoadhesion strength associated with whey protein denaturation is considered to derive from interactions associated with hydrogen bonding and disulphide bridges [130]. Furthermore, a recent review [135] investigated interactions between saliva and food proteins (focusing both on whey proteins and non-whey proteins) and suggested electrostatic interactions between positively charged food proteins and negatively charged regions of mucin as a likely mechanism. However, as noted above, there could be other relevant mechanisms involved, as proteins (including β-lactoglobulin) would remain positively charged at the neutral pH within the oral cavity [131]. More broadly, Celebioglu et al. concluded that both hydrophobic and hydrophilic interactions may be responsible for mucin interactions with various types of food protein in varied food matrix conditions (e.g., pH dependency) [135].

The investigation of these potential causes of whey protein-derived mouthdrying requires appropriate methods and ideally should be tested within the target popula-tion. Currently, the majority of the literature, as outlined in Table 3a–c has focused on using in vivo and physiochemical analysis to understand the proposed mechanisms of whey protein-derived mouthdrying alongside collecting sensory data. Key limitations are, however, associated with these methods: (1) researchers are only able to provide correlations between potential underpinning mechanisms and sensory data, and therefore, are unable to prove relationships; (2) a lack of research involving the human mouth, apart from the oral retention method developed by our research group [89,134,136]; (3) the ongoing challenge of quantifying mouthdrying using a ‘physical measure’ at the same time as scoring mouthdrying perception within products; (4) there is no defined mouthdrying threshold test to quantify individual sensitivity; (5) few studies have explored the role of individual differences on mouthdrying using consumers. Despite mouthdrying sensations being present in different whey protein-fortified food matrices, the majority of cited studies which have investigated mouthdrying in the solid food matrices have only used sensory methods (Table 3a–c) [90,93,94]. Therefore, less is known about potential mechanisms involved compared with a WPB. It is likely that within a dry low moisture system, such as a solid food, particles could aggregate or adhere to the oral cavity, causing friction [137] resulting in subsequent mouthdrying sensations. Furthermore, the strength of the interaction could be influenced by saliva, with adhesion, friction, surface tension and salivary viscosity being considered contributing factors [137]. In addition, a previous review of mucoadhesion in food systems suggested mucoadhesion strength could potentially be increased within a solid model from food product absorbing water from the oral cavity, promoting interactions, leading to swelling and spreading, as well as strengthened mucoadhesion [138]. Similarly, as alluded to by Celebioglu et al., hydrophobic and hydrophilic interactions [135] could also be relevant within a solid model, for example causing mouthdrying by causing poor dispersion between whey protein and saliva.

Strategies to reduce mouthdrying have been previously investigated with limited success. For example, Withers et al. tested different mouthdrying mitigation strategies using a sensory trained panel by adding sucrose (3% wt/wt), modulating viscosity by adding a starch thickener (1.8% wt/wt) and increasing fat levels by using both sunflower oil and milk fat (2% wt/wt), and concluded that all these strategies had minimal effect on mouthdrying in dairy beverages at the tested levels [122]. This highlights the challenges associated with suppressing mouthdrying and a need to understand better the potential mechanism involved in mouthdrying to enable improved mitigation strategies to be developed [122].

In summary, addressing and understanding the proposed causes of mouthdrying is important to increase the enjoyment derived from products and subsequent compliance. Texture has a key role in food preferences and learning from astringency related oral drying can provide useful insights into whey protein-derived mouthdrying.

### 4.2. Mucoadhesion and Mouthfeel Perception

**Figure 3:**
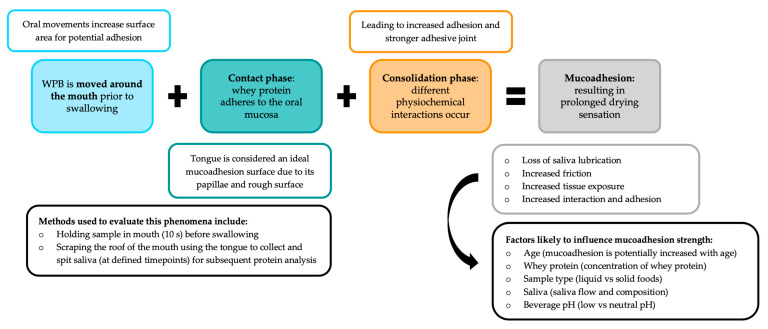
Proposed mucoadhesion mechanism of neutral pH whey protein beverages (WPB) [89,128,134,136,138,144,147].

There is a growing interest in the mucoadhesion phenomenon and its associated prolonged ‘oral exposure’, which may influence sensory perception [138]. Our research group has proposed mucoadhesion to be the probable cause of whey protein-derived mouthdrying particularly in beverages at near-neutral pH. A proposed WPB mucoadhesion mechanism is outlined in Figure 3.

Mucoadhesion is a concept that has been well utilised in drug delivery systems due to its ability to enhance retention at mucosal membranes [144,145,146,147] and has more recently been considered in a food context [138]. Mucoadhesion can be simply described as the adhesion of materials to mucosal membranes (moist surfaces lining the walls of different body cavies) [147]. Mucoadhesion can result from different physicochemical interactions, such as hydrogen bonding, hydrophobic interactions, electrostatic interactions, Van der Waals forces and disulphide bridges [144,148,149]. Mucoadhesion can be explained based on different theories, for example, wetting, mechanical, electronic, diffusion, dehydration and adsorption [147]. Two different stages have been cited in establishing mucoadhesion [144,147]. The first is a contact phase which can occur from the adhesion of a material (e.g., whey protein) to the mucosal membrane (oral mucosa), resulting in spreading and swelling) [144,147]. The second is a consolidation phase resulting from physicochemical interactions, which lead to stronger adhesion [144,147]. Mucoadhesion has often been measured using physical techniques (rheological, optical and spectroscopic) and in vivo methods (tensile, rotating disc, flow-through, tribology and oral retention) [89,128,134,136,147].

Mucoadhesion is considered in the context of this review to be the binding or sticking of whey proteins to the oral cavity (cheeks, gums and tongue) [86]. In order to measure such adhesion to the oral cavity within humans, our research group developed an oral retention method [134,136]. This method enables researchers to measure the amount of protein retained in the mouth over time by measuring protein concentration in saliva samples [134]. However, the key limitation of this method has related to a very small subject sample size and the absence of a non-protein source control. More recently, Norton et al. validated the oral retention method by establishing that WPB consumption significantly increased protein content in saliva samples post beverage, compared with a non-protein control (whey permeate beverage) using a group of younger consumers [89]. Furthermore, factors such as saliva flow, composition and viscosity are considered to influence retention of samples [136]. Accordingly, it is proposed that a reduced saliva flow could lead to greater mucoadhesion as a result of increased tissue exposure, adhesion and interactions from proteins within the oral cavity [134]. Recent work by our research group highlighted that reduced salivary flow rate correlated with increased mucoadhesion; however, differences in saliva flow had no significant influence on mouthdrying perception [89]. Therefore, we conclude the need for further research in this area involving more sensitive salivary flow rate methods, as well as including the rating of mouthdrying perception within such methods (a key limitation as alluded to in Table 3a–c) to enable better correlations with mucoadhesion.

The extent of mucoadhesion within older adults is relatively unknown. However, it is proposed that mucoadhesion is likely to be strengthened within an ageing population as (a) sensitivity to mouthdrying can increase with age [143] and (b) salivary flow rates can decrease with age [101]. Recently, we investigated this phenomenon in 84 consumers (42 younger adults aged 18–30 years and 42 older adults aged 65 years or over) [89]. Older adults had significantly increased protein concentration in saliva samples post WPB consumption, regardless of the extent of whey protein heat treatment, compared with younger adults [89]. This suggests mucoadhesion increases with age and could result in a prolonged drying sensation; however, this latter point needs further proof. Understanding the potential mechanisms involved in whey protein-derived mouthdrying will be key to ensure products are optimised so as to ensure the benefits associated with consumption of whey protein-fortified products are achieved by older adults.

In summary, mucoadhesion is a relatively new area within mouthfeel perception and early indications suggest mucoadhesion has a role as a potential cause of mouthdrying. However, this is yet to be proven, and therefore, future work should address this phenomenon, as well as identifying whether mucoadhesion is present in different food models and considering the role that individual differences may have on mucoadhesion.

## 5. Age and Individual Differences Likely to Influence Mouthfeel Perception

Sensory perception is considered to alter with age. The most obvious age-associated changes relate to vision and hearing, although touch and pain thresholds also increase with age [150,151]. It is well documented that taste impairments and loss of smell are commonly associated with ageing. For example, older adults have increased taste detection thresholds across all taste modalities and accordingly perceived taste perception declines with age [152]. Olfactory function also reduces with age and the combination of taste and olfactory decline can result in older adults often perceiving foods to lack flavour [25,152,153,154] (ageing and taste has been reviewed previously see Methven et al. [152]). Age has been shown to have varying effects on texture and mouthfeel perception. For example, studies have shown that older adults perceived soups as less creamy, sweet waffles as less fatty and elastic and dairy beverages as more mouthdrying compared with younger adults [143,155,156]. However, in other studies the effects of age have been less apparent, such as perceived thickness and mouthcoating of dairy beverages remaining consistent between younger and older age groups [143]. Again, in a study comparing different nut types using temporal dominance of sensations, the overall progression of dominant attributes during chewing was consistent between age groups [157]. Older adults did however select hardness as a more dominant attribute compared with younger adults [157]. This suggests some aspects of texture perception are potentially preserved with age, however, this could be attribute and product dependent. Accordingly, these changes can influence food choice, potentially making food less interesting and enjoyable, and therefore may increase the risk of poor nutritional status. Currently, less is known about how mouthfeel perception changes with age. Moreover, it has been suggested by previous authors that a greater emphasis could be placed on mouthfeel sensations to compensate for taste and smell loss in older adults [158].

### 5.1. Whey Protein-Derived Mouthdrying and Changes in Perception with Age

Surprisingly, despite multiple high protein products being available on the market and whey protein-fortified products being commonly used to improve nutritional status, these products are typically not designed with, or for, older adults. Withers et al. suggested some aspects of texture perception are influenced by age, as older adults reported greater sensitivity to mouthdrying compared with younger adults following consumption of dairy beverages [143]. This study investigated mouthdrying by comparing a heated rennet whey sample with a skimmed milk sample using a paired comparison test [143]. Only the older adults were able to distinguish the rennet sample as more mouthdrying [143]; rennet whey was proven previously to be a source of mouthdrying [133]. However, two more recent studies by our research group [89,90] have been unable to demonstrate an overall effect of age on mouthdrying in different whey protein food models using a gLMS (generalised linear magnitude scale) and VAS (visual analogue scale) [89,90]. They concluded that within a liquid model using WPBs, the potential cause of the minimal effect between age groups related to the lack of sensitivity of the gLMS compared with a paired comparison test [89]. Whereas, in the solid model, which used two different methods (a single point in time and a full portion size at home), the older adults were able to perceive the protein cakes and biscuits as more mouthdrying compared with the control versions [90]. This supported Withers et al. findings [143] but did not reach overall significance and highlighted the challenges with measuring mouthdrying within an older population and ensuring a suitable test is selected to measure such mouthdrying.

Sensory testing needs to replicate normal eating behaviour and measure changes in consumption over repeated consumption, rather than just a single sip or bite. This is especially relevant to products such as ONS, which are associated with changes in liking and mouthfeel with multiple sips [85,87,88]. This demonstrates the challenges within older adults of balancing the appropriate volume to replicate normal consumption versus sample fatigue from too many samples [38] (for a review of sensory and consumer methodology in older adults, see Methven et al. [38]). Moreover, to measure effectively changes in the perception of mouthdrying with age, it is important to ensure the methods to be used are suitable for a broad range of older adults within a test group, so as to secure useful and meaningful results.

### 5.2. Individual Differences That Could Influence Perception of Whey Protein-Fortified Products

Individuals are defined by differences that distinguish them from others and such differences can influence sensory perception. For example, consumers typically differ in physiology (such as age, biological sex, health status and associated medications, appetite, dental status, saliva flow, muscle strength, sensory acuity—including differences in taste, olfaction and oral tactile sensitivity), social factors (such as cultural and demographic groups) and preferences (such as food preferences, mouth behaviour and food neophobia) [159,160,161,162,163,164,165,166]. When designing products for older adults, individual differences are likely to influence perception. Accordingly, these differences will be explored in the following sections with a specific focus on their relevance for older adults. Table 4 highlights that individual differences, such as age, oral health, saliva and food oral processing are considered to have a role within sensory perception. However, currently the extent of the effect of such individual differences on the perception of whey protein-derived mouthdrying is relatively uncertain.

### 5.3. Food Oral Processing and Mouthfeel Perception

The oral cavity consists primarily of lips, gums, cheeks, hard and soft palates, teeth, tongue, salivary glands, orofacial muscles and mucous membranes [150,167]. The oral mucosa (three types within the oral cavity, namely lining, masticatory and specialised mucosa) is a moist soft tissue membrane lining the oral cavity providing key functions such as protection, lubrication and moistening [150,168]. Oral receptors respond to food digestion and processing, thereby leading to taste, odour, irritation and texture perceptions [160]. The mouth is considered a sensitive organ and receptors such as mechanoreceptors (touch and proprioception), which respond to tactile stimuli, are considered the most relevant for texture perception [160]. Although there is no specific texture receptor, texture is considered to be perceived by the tongue, palate and other soft tissues within the mouth [160]. For further details on oral cavity anatomy and physiology and relevant oral receptors within a food context, see references [150,160,168,169,170].

Individuals differ in their masticatory function, bite force, swallowing threshold, saliva volume and composition, oral receptors and sensitivity [159]. Therefore, it is combination of differences in food structure and individual oral physiology that cause variation in food oral processing and subsequently in sensory perception [170]. Differences in food oral processing influence perception not only of texture and mouthfeel, but also flavour, thereby affecting food choice and acceptability [170,171]. Mouth behaviour can be described as the way an individual manipulates food in their mouth and is considered to influence food choice, texture preference and satisfaction [165,166]. There are four major mouth behaviour groups: crunchers (individuals that like foods that break on biting) and chewers (those that prefer to chew foods), being considered the more predominant groups compared with suckers (those that prefer harder foods which can be sucked on) and smooshers (likers of soft foods and less mouth activity) [165,166]. Mouth behaviour (as outlined in Table 4) can also have implications for older adults. For example, a decline in dental status can influence food choice, resulting in a preference for softer foods rather than hard crunchy foods [165,166].

Food lubrication within the mouth is considered to be influenced by size and concentration of oil droplets, viscosity of saliva, protein content of saliva and properties of the particles (size, shape and hardness) within the oral fluid and surface properties of the oral mucosa and teeth [172]. The role of oral lubrication in food intake is also a consideration; therefore, manipulating oral lubrication could be particularly relevant within older adults who are at increased risk of malnutrition and their saliva flow often being reduced [173].

Understanding changes in food oral processing with age is key to improving food intake, particularly in an older adult population. For example, older adults are considered to consume foods more slowly, have increased chewing duration and reduced tongue strength compared with younger adults [162,174,175]. Teeth loss is also associated with ageing, data from the ‘Adult Dental Health Survey 2009—England’ demonstrated edentate increasing with age from 1% at 45–54 years, 5% at 55–64 years, 15% at 65–74 years, 29% at 75–84 years and 45% at 85 years and over [176]. Teeth loss is also associated with reduced masticatory abilities [177], and Steele et al. noted from a study involving 1211 adults aged 60 years or over that having 21 or more natural teeth resulted in less eating problems [178]. Mastication behaviour can influence mouth behaviour preferences, texture perception of foods and food choice and intake, thereby impacting an individual’s nutritional status [179,180]. For example, lower mucosal moisture has been associated with reduced and poor chewing capacity in older adults [181].

In summary, food oral processing is considered to play an important role in determining food choice and acceptance, with age-related changes likely to impact this further. Changes in food oral processing are likely to impact perception and acceptance of protein-fortified foods. Overall, an understanding of the differences between age groups and their sensory sensitivity will assist in the provision of more suitable food products to match the needs of older adults.

### 5.4. Differences in Saliva Flow with Age and Their Potential Effect on Mouthfeel Perception

Saliva is a viscoelastic solution, consisting of approximately 99.5% water, with the remainder (~0.5%) being proteins, enzymes, electrolytes and nitrogenous products [150,182,183,184]. Saliva performs a key role in the maintenance of oral health, as well as enabling taste, providing a buffer capacity and mineralisation, aiding digestion and preventing tooth decay, as well as being a lubricant and having antimicrobial functions [182,183,184].

Saliva-related diseases can negatively impact oral health, quality of life, dietary habits and nutritional status [185]. For example, xerostomia (dry mouth) is a syndrome involving an absence of saliva and results in eating difficulties, tooth decay and oral candida infection and its prevalence is considered to increase with age [186]. Hyposalivation (reduced saliva flow) is typically cited as <0.1 and 0.5 mL/min for unstimulated saliva and stimulated saliva flow, respectively [185,187,188]. It correlates with adverse health outcomes, as well as reduced taste perception, chewing and swallowing difficulties [185,187,188]. Common causes of hyposalivation include medication, dehydration and disease [185]. Prevalence of dry mouth within an older population is considered between 12–39% and increases with age [102].

**Figure 4:**
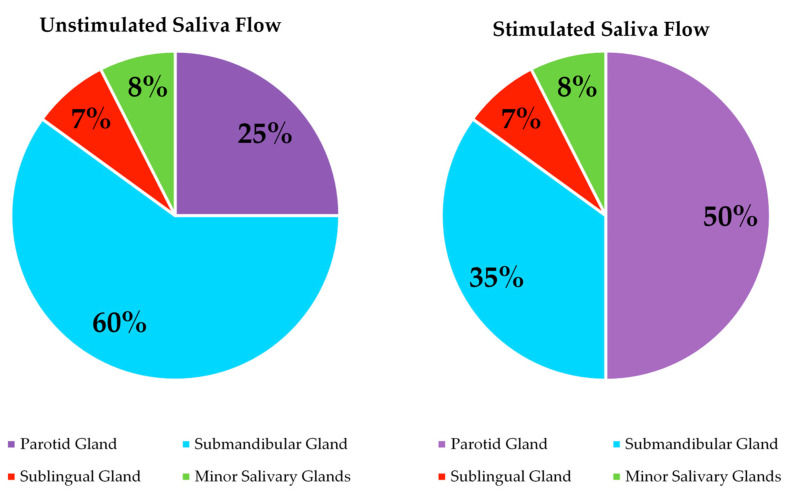
Saliva flow contribution from salivary glands [191].

There is evidence of age-related changes in saliva. For example, a review by Xu et al. highlighted salivary changes with age, supporting reduced saliva flow, changes in calcium and mucin content and increased ionic concentration influencing the quantity and quality of saliva [189]. A meta-analysis involving 47 studies concluded significantly reduced salivary flow rates in older adults, and this reduction was not considered to be related to use of medication [190]. Vandenberghe-Descamps et al. demonstrated that healthy older adults had 38.5% and 38% lower resting and stimulated salivary flows respectively when compared with younger adults and the results were independent of medication and dental status [101]. The acinar cells are considered to degenerate with age and can influence salivary flow rates [191]. Affoo et al. indicated a gland specific reduction in salivary flow rates in older adults and highlighted that the parotid gland and the minor glands are potentially less influenced by age [190]. An overview of saliva flow contributions from salivary glands [191] is outlined in Figure 4. The submandibular gland, which contributes 60% of unstimulated saliva production, has an increased sensitivity to metabolic and physiological changes, which is a proposed cause of greater changes seen in unstimulated saliva flow with age compared with stimulated saliva [191]. Accordingly, a reduced saliva flow is considered an issue and is commonly associated with decreased lubrication, protection, oral clearance, mucosal surfaces hydration and coating abilities within the oral cavity [192,193,194,195]. It is, therefore, likely to contribute to changes in food habits, further negatively impact nutritional status and alter sensory perception (Table 5). However, as stimulated saliva flow is potentially less influenced by age [190], this may minimise changes from saliva flow in response to food consumption and subsequent sensory perception.

Food breakdown and perception of taste, flavour and texture of foods are all influenced by saliva, which affects the eating process and food intake [180,197]. Saliva provides a key role in our eating experience, with food oral processing and perception both being influenced by a number of food and saliva interactions as outlined in Table 5 [196]. Without saliva, food deformation, breakdown and destabilisation would be negatively influenced, along with food perception and swallowing [196]. In a food bolus, food particles are incorporated with saliva into something safe to swallow, and this process is in most cases considered automatic [198]. However, bolus formation and swallowing can provide additional risks in an older adult, thereby affecting an individual’s food choice and intake [198]. Additionally, these processes are considered to be influenced by the surface coating of food particles, particle size distribution and saliva incorporation, with moisture content and type of food structure also influencing the volume of saliva required [196,198].

In terms of sensory perception, unstimulated saliva provides background taste, whilst stimulated saliva is part of the mechanical process during eating and can increase salivary flow rates by 5–50 times, with more than 50% secreted from parotid glands [190,199]. As highlighted in Table 5, saliva is likely to contribute negatively to mouthfeel perception and could impact the perception of whey protein-derived mouthdrying. The spinnbarkeit test relates to the stringiness of saliva and its adhesion properties within the mouth; saliva provides lubrication and protection, both of which are considered important for sensory perception [200,201]. Altered or reduced viscoelasticity can impact mouthfeel perception, with viscoelasticity being noted to reduce with age [200,201]. Furthermore, it has been suggested that an altered aroma perception in older adults could be caused by reduced stimulated saliva flow [202]. There are challenges associated with understanding the role of saliva on subsequent perception, and these are partly due to methodology limitations (as highlighted in a recent review by Munoz-Gonzalez et al. [188]). Typically, studies have grouped volunteers into low or high saliva flow, often resulting in minimal effects on sensory perception [28,89,90].

In summary, there is a clear need to understand how saliva can impact sensory perception and consumption of foods in older adults. The influence of saliva and age-related changes in saliva on the sensory perception of foods, and specifically protein-fortified products, needs further investigation.

## 6. Conclusions

This review highlighted that individual differences (such as age, appetite, dental status, saliva flow, detection thresholds to sensory stimuli, cultural differences and preferences) could influence whey protein-derived mouthdrying, which in turn impacts the eating experience. Protein needs are considered to increase with age and protein consumption is associated with numerous benefits. More specifically, whey protein is commonly fortified into products due to its associated functional benefits. However, such products can elicit mouthdrying, which is considered to hinder consumption and acceptance. Therefore, improvements in such products are key to increasing liking and reducing wastage. Furthermore, mouthdrying is considered to increase with age, and despite previous investigations, the causes of whey protein-derived mouthdrying are currently not fully understood. Further research is needed to understand these, with mucoadhesion currently being a proposed, but as yet to be proven, cause. In addition, more research is needed into potential mitigation strategies (such as using fat, sucrose or adjusting viscosity) to modulate mouthdrying and their subsequent influence on consumer acceptance. Despite mouthdrying being present in both a liquid and solid food model, research has mainly focused on WPB mechanisms rather than solid model mechanisms; therefore, future research should look to address this gap within the literature. Individual differences, such as age, oral health, saliva and food oral processing, are considered to have a role within mouthfeel perception. However, currently, the effect of such individual differences on mouthdrying and mucoadhesion is relatively uncertain. If taking account of age and individual differences could lead to increased protein consumption by tailoring whey protein-fortified products to meet individual needs, then this could significantly improve nutritional status in older adults and help to reduce their susceptibility to malnutrition and sarcopenia.

## Figures and Tables

**Table 1:** Suggested risk factors for malnutrition (adapted from [12,13,14]).

Social	Physical	Medical	Psychological
Living and eating alone	Physical disabilities	Swallowing difficulties (dysphagia)	Anxiety
Poverty	Reduced appetite	Eating disorders	Depression
Difficulty in shopping or preparing food	Poor dentition	Medication	Dementia
Limited nutrition knowledge and cooking skills		Conditions leading to reduced appetite and absorption/utilisation of nutrients	Bereavement

**Table 2:** Commonly proposed causes of whey protein beverage (WPB) derived mouthdrying adapted from Norton et al. [89] and associated limitations.

Proposed Cause	WPB Model ^1^	Description	Limitations
pH of WPB	WPC [86,122], WPI [124,139], WPI, β-LG and LF [125,126]	o Low pH can cause precipitation of the protein	o There is evidence of mouthdrying from WPB at both low and neutral pH
Saliva and protein interactions	β-LG [123], WPI [124,127], WPI, β-LG and LF [125,126]	o Perception of mouthdrying has links to saliva and protein interactions	o Studies have used in vivo analysis mixing human or artificial saliva with whey proteins, but this requires sensory analysis to correlate instrumental data with mouthdrying
Reduced lubrication from saliva	β-LG [128]	o Increased friction within the oral cavity from reduced lubrication	o Using instrumental analysis (such as tribology) to predict in-mouth experiences, but this requires sensory analysis to correlate instrumental data with mouthdrying
Adhesion and binding properties	WPC [89,134], β-LG and LF [140], WPI [130], β-LG [131]	o Whey proteins binding to oral epithelial cells, proteins remaining on surfaces, mucoadhesive properties, increased oral retention and whey protein adhering to the oral cavity	o In vivo, animal models, small subject size, without a non-protein source control, but this requires sensory analysis to correlate instrumental data with mouthdrying
Heating time	WPC [86], RW [133]	o Mouthdrying is considered to increase with product heating time, potentially due to protein denaturation	o Mouthdrying is present in samples without heat treatment, albeit at lower levels, so this cannot be the sole cause

^1^ Whey protein beverage (WPB) model: whey protein concentration (WPC), whey protein isolate (WPI), β-lactoglobulin (β-LG), lactoferrin (LF) and rennet whey (RW).

**Table 3:** (**a**) Sensory methods commonly used to investigate whey protein-derived mouthdrying. (**b**) Physiochemical analysis commonly used to investigate whey protein-derived mouthdrying. (**c**) In vivo analysis commonly used to investigate whey protein-derived mouthdrying.

**(a)** Sensory methods commonly used to investigate whey protein-derived mouthdrying.			
**Method**	**Food Matrix**	**Description**	**Limitations**
**Sensory methods using trained panel or consumers. Key limitation: unable to explain the cause of mouthdrying**			
Descriptive analysis using a trained sensory panel ^1,2^	Cakes and biscuits [90], WPB [83,86,91,92,123,124,125,126,140,141,142], rye bread and cream cheese [94]	o Provides an objective sensory measure of mouthdrying	o Studies have used different methods (such as Spectrum^TM^ and QDA^TM^), scored differing numbers of attributes (2 to 36) and there are potential issues with providing a standard mouthdrying reference to ensure consistency across studies
Threshold using a trained sensory panel ^1^	WPB [123,139,140]	o Evaluates mouthdrying intensity strength compared with protein concentration	o Studies have rated mouthdrying intensity using different methods (for example: 0–5 and 0–7 scales, Spectrum^TM^ and scalar scoring), different types of whey protein beverages and studies have used varying number of panelists (7–12 panelists)
Sequential profiling and time intensity methods using trained sensory panels ^1^	WPB [86,122,123,124]	o Sequential profiling measures changes in sensory attributes with repeated consumption and time intensity provides data on time, duration and intensity of mouthdrying	o Typically, sequential profiling methods have not solely focused on mouthdrying and there are also potential issues with providing a standard mouthdrying reference to ensure consistency across studies
Sensory methods using consumers ^1,2^	WPB [83,89,91,92,143], cakes and biscuits [90], muffins [93], rye bread and cream cheese [94]	o Provides feedback on products using the target consumer population. Common methods to evaluate mouthdrying include focus group sessions, 9-point hedonic liking, Just-About-Right (JAR), generalised linear magnitude scale (gLMS), visual analogue scale (VAS) and two-alternative forced choice test (2-AFC).	o Limited studies have tested mouthdrying using consumers and there are potential issues with test sensitivity of methods used. Carter et al. noted consumers are untrained and potentially less able to quantify mouthdrying objectively [46].
^1^ Refers to studies using a whey protein liquid model (whey protein beverage: WPB); ^2^ refers to studies using a whey protein solid model.			

**(b)** Physiochemical analysis commonly used to investigate whey protein-derived mouthdrying.			
**Method**	**WPB Model**	**Description**	**Limitations**
**Physiochemical analysis. Key limitation: requires sensory data to provide correlations**			
Taste sensor ^1^*	WPI, PWP, aPWP [139]	o Measures the change in membrane potential as a result of adsorption	o Analysis has been carried out in low pH WPBs; therefore, this method may not be suitable for neutral pH WPBs
Turbidity ^1^*#	β-LG [123], WPI [124], β-LG and LF 126]	o Measures aggregation of protein and saliva	o Saliva has been mixed artificially with whey protein and this may differ to saliva samples collected post beverage consumption o Saliva samples in the referenced studies were only collected from 2–5 volunteers; however, saliva is considered to vary between individuals o Turbidity in isolation is unlikely to explain the cause of mouthdrying
Electrophoresis analysis ^1^*#	β-LG and LF [125,126]	o Determines protein composition using SDS-PAGE (sodium dodecyl sulfate polyacrylamide gel electrophoresis)	o As for turbidity: saliva has been mixed artificially with whey protein; saliva samples only collected from 2–5 volunteers
Dynamic Light Scattering ^1^*#	WPC [86], β-LG and LF [126]	o Measures the size and distribution of protein and/or with saliva	o As for turbidity: saliva has been mixed artificially with whey protein o Particle size in WPB increases with heating time, however, mouthdrying is also present in unheated WPBs. Therefore, particle size in isolation is unlikely to explain the cause of mouthdrying
Zeta potential ^1^*#	WPC [86], β-LG and LF [126,140]	o Measures electrostatic interactions of protein, with or without saliva	o Bull et al. identified within a neutral pH that WPBs (samples varying in levels of heat treatment) had similar zeta potential scores, therefore proposed mouthdrying in this study was not related to electrostatic interactions and proposed other mechanisms could be involved. However, saliva was not collected in this study [86].
Portable infrared spectrometer ^1^*	WPI, WPC, WPH [141]	o Predicts mouthdrying in low pH WPB	o This method was only tested in low pH WPBs; therefore, this method may not relate to mouthdrying from neutral pH WPBs
Tribology ^1^*	β-LG [128]	o Measures friction and lubrication	o In some conditions (i.e. increasing protein concentration from 0.5 to 4%) sensory results were unable to correlate with tribology data
^1^ Refers to studies using a whey protein beverage (WPB) model (whey protein isolate (WPI), process whey protein (PWP), acidic process whey protein (aPWP), whey protein concentration (WPC), whey protein hydrolysate (WPH), β-lactoglobulin (β-LG) and lactoferrin (LF); ^2^ refers to studies using a whey protein solid model; *denotes studies using a low pH WPB model; #denotes studies using a neutral pH WPB model.			

**(c)** In vivo analysis commonly used to investigate whey protein-derived mouthdrying.			
**Method**	**WPB Model**	**Description**	**Limitations**
**In vivo analysis. Key limitation: requires sensory data to provide correlations**			
Saliva flow ^1^*	β-LG [123]	o Evaluates saliva flow following different stimulants and relating this to whey protein-derived mouthdrying	o Studies have been limited by the number of saliva samples which can be collected within one session and this referenced study was limited by a relatively small sample size (10 volunteers) with a gender imbalance (2 males and 8 females)
Animal models ^1^#	β-LG [131]	o Measures the adhesion of proteins to porcine oral mucosa tissue	o Methods need to be adapted to enable human investigation
Oral retention ^1^#	WPC [89,134]	o Measures the protein remaining in saliva samples post beverage consumption	o Previous limitations were small subject size and no non-protein control; more recent limitations include the link between mucoadhesion and mouthdrying within the same method have not been investigated
Dynamic in vivo models ^1^*	WPI [127]	o Aims to replicate in-mouth beverage consumption by measuring whey protein and saliva interactions	o Models were estimated based on limited data from the literature, therefore may not fully reflect individual variability
^1^ Refers to studies using a whey protein beverage (WPB) model (whey protein concentration (WPC), whey protein isolate (WPI), β-lactoglobulin (β-LG); ^2^ refers to studies using a whey protein solid model; * denotes studies using a low pH WPB model; # denotes studies using a neutral pH WPB model.			

**Table 4:** Relevant individual differences likely to influence perception of whey protein-derived mouthdrying in older adults (↑ increases with age; ↓ decreases with age; n/a not applicable).

Category	Factors	Effect of Age	Effect on Mouthdrying	Food Matrix	Methodology Limitations
Physiology	Age ^1^*	n/a	o Whey protein fortified products cause mouthdrying which may be influenced by age	WPB [89,143], cakes and biscuits [90]	o Inconsistent results between studies could result from differences in test sensitivity used (for example, paired comparison test vs generalised labelled magnitude scale)
	Appetite ^1,2^*#†	↓	o ONS and whey protein-fortified products can increase perceived thirst, reduce hunger and prospective consumption	Cupcakes [90], ONS [87,88]	o Self-report using visual analogue scale. Appetite was not measured at subsequent meals
	Dental status ^1,2^*†	↓	o Poor dental status could make consumption of solid foods more difficult and therefore negatively impact product liking	Cakes and biscuits [90], meat and cereal [28]	o Self-report questionnaire or limited oral parameters measured
	Saliva flow ^1,2^*#	↓	o Saliva flow can decrease with age, however whether this influences subsequent perception is relatively unclear	WPB [89,123], cakes and biscuits [90], meat and cereal [28]	o Volunteers may have been too healthy to demonstrate an effect of saliva flow
	Detection thresholds to sensory stimuli ^1^#	↑	o Detection thresholds for many stimuli (such as tastants and volatile compounds) increase with age and perception increases (at different rates depending on the stimuli) with stimuli intensity. Studies to date suggest that perceived mouthdrying initially increases with protein concentration until a plateau is reached.	WPB [83,123,139,140]	o No defined mouthdrying threshold method has been developed
Social	Culture ^2^*	n/a	o Cultural groups have different food oral processing behaviour and this could influence food choice and mouthfeel perception	18 different food products varying in physical properties [162] carrot, cheese and sausage [163]	o Only limited populations have been studied (for example Dutch nationality and Caucasian ethnicity compared with Chinese nationality and Asian ethnicity)
Preferences	Food preference and neophobia ^2^‡	No set direction	o Food preferences and neophobia could influence compliance with ONS and whey protein-fortified products	n/a [161,164]	o Self-report questionnaire
	Mouth behaviour ^1^*	Not known	o Mouth behaviour could influence texture perception of whey protein-fortified products and may alter with age	Cakes and biscuits [90]	o Self-report questionnaire

^1^ Refers to studies using whey protein food matrices; ^2^ refers to factors that may influence whey protein derived mouthdrying but have currently not been investigated within a whey protein food matrix. Study type: * younger adults (18–35 years) and older adult (over 65 years) study; # younger adults only (20–60 years); † older adults only (87: 60–75 years; 28: over 65 years); ‡ other: (161: children aged 9–12 years and parents; 164: n/a review paper). All volunteers considered healthy unless otherwise stated.

**Table 5:** Summary of proposed food and saliva interactions and effect on sensory perception, as suggested by Mosca and Chen [196].

Proposed Mechanism	Description	Sensory Perception
Surface coating and wetting	o This ensures lubrication, food breakdown, bolus formation and safe swallowing	o Insufficient saliva can result in drying sensations
Colloidal interactions	o Colloidal food products such as beverages and emulsions can interact with saliva causing destabilisation	o Texture and mouthfeel attributes
Complexation	o Reduction in saliva lubrication and increased friction	o Mouthdrying and astringency sensations
Enzymatic breakdown	o Rheological properties changes from amylase activity and macromolecules partial hydrolysis	o Texture and flavour perception
Binding of aroma compounds	o Saliva dissolves tastants and binds aroma	o Flavour perception
